# POOR SLEEP QUALITY IS RELATED TO DECREASED WHITE MATTER INTEGRITY IN BRAIN NOCICEPTIVE PATHWAYS IN OLDER ADULTS

**DOI:** 10.1093/geroni/igz038.1343

**Published:** 2019-11-08

**Authors:** Anna R Egbert, Ryan S Falck, John R Best, Linda Li, Lynne Feehan, Teresa Liu-Ambrose

**Affiliations:** University of British Columbia, Vancouver, British Columbia, Canada

## Abstract

Poor sleep quality, decreased physical activity (PA) and increased sedentary behavior (SB) are common characteristics of older adults. Notably, these factors play an important role in brain health. We examined the relationship between sleep quality, PA, SB and brain white matter integrity (WM) in older adults with osteoarthritis (OA). We retained data on 16 participants (mean age 60, SD=7.7) from a larger Monitor-OA cohort recruited from Metro Vancouver, BC, Canada. Sleep efficiency and duration, amount of time spent on PA and SB daily over a period of one week was acquired with an objective measure – the multi-sensor monitor SenseWear Mini which integrates tri-axial accelerometer data, physiological sensor data and personal demographic information. Brain WM tractography was calculated from fractional anisotropy data obtained with diffusion weighted magnetic resonance imaging. Voxelwise group-level statistics examined the effects of our variables of interest on the integrity of brain WM tracts while controlling for participants age. We found that lower sleep efficiency was related to decreased integrity in WM tracts of frontal, temporal lobes, precuneus and thalamus (Bonferroni corrected p<0.05). Shorter sleep was related to lower WM integrity in frontal regions, posterior cingulate and insula radiations (Bonferroni corrected p<0.05). No significant effects were noted for PA or SB. The identified brain regions are involved in sleep processes but further overlap with the nociceptive brain network. Our findings suggest that neural mechanisms related to sleep disturbance may also involve pain-related processing in older adults.

